# Single‐nuclei sequencing in ovariectomized rat hippocampus identifies estrogen‐sensitive brain cell sub‐populations and pathways

**DOI:** 10.1002/alz70855_101089

**Published:** 2025-12-23

**Authors:** John W. McLean, Tian Wang, Yuan Shang, Roberta Diaz Brinton

**Affiliations:** ^1^ University of Arizona, Tucson, AZ, USA

## Abstract

**Background:**

Estrogen is a master regulator of systems biology in the female brain, coordinating metabolic, immune, and neuronal homeostasis. Loss of estrogen during menopausal transition is associated with declining brain glucose metabolism and increasing neuroinflammation, which may contribute to neuronal vulnerability and a doubled lifetime risk of Alzheimer's disease (AD) in women. However, neuronal and glial cell type‐specific responses to estrogen deficiency in the hippocampus, a brain region critically affected in AD, is incompletely understood.

**Method:**

To identify hippocampal cells and pathways affected by estrogen loss, 5‐month‐old female Sprague Dawley rats were randomly assigned to sham ovariectomized (SHAM), ovariectomized plus vehicle (OVX), or OVX with 17β‐estradiol (OVX+E2) and sacrificed 5 weeks post‐surgery.

Nuclei isolated from dorsal hippocampus were pooled from two biological replicates per treatment group. A 10X Genomics Chromium Single Cell 3’ kit was used to generate a single‐nuclei cDNA library for each condition. Libraries were sequenced on an Illumina NovaSeq6000, and demultiplexed FASTQ files were analyzed using CellRanger 7.1.0 and Loupe Browser 8.0. After a quality‐control step filtering out nuclei with >5% mitochondrial reads, established cell type‐specific marker genes allowed annotation of distinct clusters of major brain cell types including excitatory and inhibitory neurons, astrocytes, oligodendrocytes, oligodendrocyte precursor cells (OPCs), microglia, and endothelial cells.

**Result:**

Single‐nuclei transcriptomics identified reduced cell percentage of a neuron cluster (Exc1) expressing *Slc17a6* (vGlut2) in OVX hippocampus (10%), which was partially rescued in OVX+E2 (17%), relative to SHAM (37%). Additional subpopulations affected included other inhibitory and excitatory neuron clusters, as well as oligodendrocytes. Bulk analysis identified KEGG pathways altered in OVX condition, including antigen processing, cholesterol metabolism, insulin resistance, and glutamatergic synapse pathways. Gene Set Enrichment Analysis of differentially expressed genes within estrogen‐sensitive neuronal subpopulations indicated a reduction in oxidative phosphorylation pathways in OVX compared to both SHAM and OVX+E2.

**Conclusion:**

These findings extend previous reports of estrogen loss affecting bioenergetics, specifically oxidative phosphorylation, as well as neuroinflammation and synaptic function. Ongoing studies aim to identify further cell‐type specific pathway contributions. Outcomes will advance an understanding of brain cell‐specific responses to estrogen loss and help identify specific drivers of brain vulnerability in the estrogen‐deficient female brain.

